# Are patients more adherent to newer drugs?

**DOI:** 10.1007/s10729-020-09513-5

**Published:** 2020-08-08

**Authors:** Katharina E. Blankart, Frank R. Lichtenberg

**Affiliations:** 1 Columbia Business School, Columbia University, New York, NY USA; 2 Present Address: Faculty of Economics and Business Administration, University of Duisburg-Essen and CINCH – Health Economics Research Center, Campus Essen, Berliner Platz 6-8, 45127 Essen, Germany; 3 Hamburg Center for Health Economics, University of Hamburg, Hamburg, Germany; 4 National Bureau of Economic Research, Cambridge, MA USA

**Keywords:** Medicines possession ratio, Innovation, Vintage, Promotion, Productivity in health care, I12 Health Behaviour, L65 Industry Studies: Biotechnology, C31 Cross Sectional Models

## Abstract

The annual preventable cost from non-adherence in the US health care system amounts to $100 billion. While the relationship between adherence and the health system, the condition, patient characteristics and socioeconomic factors are established, the role of the heterogeneous productivity of drug treatment remains ambiguous. In this study, we perform cross-sectional retrospective analyses to study whether patients who use newer drugs are more adherent to pharmacotherapy than patients using older drugs within the same therapeutic class, accounting for unobserved heterogeneity at the individual level (e.g. healthy adherer bias). We use US Marketscan commercial claims and encounters data for 2008–2013 on patients initiating therapy for five chronic conditions. Productivity is captured by a drug’s earliest Food and Drug Administration (FDA) approval year (“drug vintage”) and by FDA” therapeutic potential” designation. We control for situational factors as promotional activity, copayments and distribution channel. A 10-year increase in mean drug vintage is associated with a 2.5 percentage-point increase in adherence. FDA priority status, promotional activity and the share of mail-order prescription fills positively influenced adherence, while co-payments had a negative effect. Newer drugs not only may be more effective in terms of clinical benefits, on average. They provide means to ease drug therapy to increase adherence levels as one component of drug quality, a notion physicians and pharmacy benefit managers should be aware of.

**Electronic supplementary material:**

The online version of this article (10.1007/s10729-020-09513-5) contains supplementary material, which is available to authorized users.

**Highlights**

- We perform comparisons within patients to analyze the role of drug quality in medication adherence
- We designed an integer linear program that can only be solved for small instances.
- We consider co-payments, promotion and patient heterogeneity as additional factors.
- We use the concept of vintage as measured by the US Food and Drug Administration’s year of market approval as an indicator of a drug’s average quality; later-vintage drugs are hypothesized to have higher average quality than earlier-vintage drugs.
- Prescribers and pharmacy benefit managers should be aware that patients tend to be more adher-ent to newer drugs within a therapeutic class when making prescription choices.

## Introduction

In chronic diseases, biomedical innovation has been shown to contribute to improving health outcomes of populations [1]. Almost 90% of private biomedical R&D is performed by biopharmaceutical companies [2], and government-funded R&D also plays an important role in drug development [3]. Successful drug therapy not only depends on the type of drug agent used. Equally if not more important is whether patients use the medicines prescribed as advised. Outside clinical trials, low adherence is widespread [4–6]. Moreover, non-adherence is recognized as a significant source of waste. The annual preventable cost from non-adherence in the US health care system has been estimated at around $100 billion [7, 8]. Non-adherence accounts for about half of the potentially avoidable cost of inappropriate medication usage.

Adherence levels may be improved by interventions that manage drug therapy and providers being able to anticipate potential non-adherence [9, 10]. However, overall, the evidence about whether interventions that target medication non-adherence are favorable in terms of health outcomes and cost is ambiguous [11–14]. Many interventions had unfavorable cost-effectiveness ratios [15] or were dominated. Best-practice interventions have yet to be identified [16].

Outside evaluation of programs to manage adherence, associations between medication adherence, health outcomes and cost of health care have been shown [17–22]. Higher adherence levels are associated with reduced hospital admissions [23, 24] and reduced mortality [25, 26] from selected chronic diseases such as diabetes and hypertension. Although higher adherence levels may cause increases in initial pharmaceutical costs, overall health care costs may decline due to the health services cost offset [27]. Reasons for medication non-adherence have been found to fall into several categories: the health system, the condition, patient characteristics, the therapy, and socioeconomic factors [28].

One potential determinant of adherence is the extent of innovation embodied in, or ‘quality’ of, the drug agent; this is the subject of this study.^1^ This factor that captures productivity aspects of drug treatment has not been considered in the vast literature on the determinants of medication adherence. Evidence exists that pharmaceutical innovation has contributed at significant levels to increase longevity [29]. Also, diseases subject to more rapid pharmaceutical innovation have experienced larger declines in work- and school-loss days [30]. The theoretical and empirical economic literature on the role of technological change in economic growth and the marketing literature on the role of product quality and repurchasing behavior provide the conceptual framework to analyze the relationship between the vintage of a drug and average drug quality to capture productivity and the subsequent relationship between drug quality and repurchase behavior, reflected by medication adherence.

The objective of this study is to investigate whether patients who use newer (i.e. later vintage) drugs are more adherent to pharmacotherapy than patients using older drugs within the same therapeutic class, ceteris paribus. We will control for potentially confounding factors of productivity of drug treatment, most importantly unobserved patient heterogeneity and promotional activities.

## The impact of drug vintage on medication adherence

Our basic hypothesis is that adherence is positively related to the quality of drugs used to treat a condition. Quality is undoubtedly multidimensional, and we do not believe that there is a single, widely-accepted measure of drug quality. However, many economists have argued that, in the case of research-intensive goods like pharmaceuticals, newer (later-vintage) goods tend to be of higher quality than older goods [31–34]. Solow was the first to hypothesize that most technological change is embodied in new goods [34]. To benefit from technological change, one must use later-vintage goods or services. Therefore, product vintage has become a widely-accepted *determinant* of average product quality: [32, 34–36].^2^ For example, as noted by Jovanovic and Yatsenko [35], in “the Spence–Dixit–Stiglitz tradition […] new goods [are] of higher quality than old goods.” Grossman and Helpman [32] developed “a model of repeated product improvements in a continuum of sectors; each product follows a stochastic progression up a quality ladder.” Solow [34] argued that “improvements in technology affect output only to the extent that they are carried into practice […] by the replacement of old-fashioned equipment by the latest models [...]”. Aghion and Howitt [33] state that “[t]echnological progress, the mainspring of long-run economic growth, comes from innovations that generate new products, processes and markets.” These “[i]nnovations in turn are the result of deliberate research and development activities that arise in the course of market competition. These Schumpeterian observations constitute the starting point of that branch of endogenous growth theory built on the metaphor of quality improvements…” Using these insights allows developing “a growth model with quality-improving innovations” to analyze microeconomic issues such as performed in this study.

Translating the vintage concept to pharmaceuticals, newer agents may be more clinically effective on average compared to older agents. In addition, newer agents may have characteristics that improve the ease of drug administration and have better side-effect profiles, all of which could improve adherence levels of patients. Less frequent dosing, one feature of drug quality, has been shown to improve adherence [38]. When patients do not need to take their medicines as often, the ease of medication use is improved. However, characteristics of a drug other than dose frequency may influence the propensity of patients who use the drug to be adherent. Of note is that key decision-makers of drug therapy (patients and providers alike) may not be aware of a drug’s vintage such that the decision to use a drug is typically not made based on a drug’s newness. However, drug vintage is an important proxy for a drug’s average quality in terms of effectiveness and ease of use which has an impact on adherence.

Regarding the quality ladders in biomedical innovation, some authors have argued that most new drugs have not provided real advances over existing drugs. This means that the average quality of newer drugs is not necessarily higher the average quality of older drugs. On the basis of limited evidence, a systematic review of new drugs for over 100 indications approved by the US Food and Drug Administration found that superior efficacy on clinical outcomes was confirmed in less than 10% of cases [39]. Wieseler et al. state that the proportion of true innovation is less than 15% [40]. For cancer drugs, evidence suggests that most drugs approved by the European Medicines Agency had been approved with no evidence of clinically meaningful benefit for patient relevant outcomes in the period 2009–2013 [41]. This notion would suggest that there should be little or no correlation between a drug’s vintage and changes in health. However, there are a number of studies that have demonstrated a substantial impact of pharmaceutical innovation, measured by changes or differences in drug vintage, on health outcomes [1, 29, 30]. For example in US cancer patient, Lichtenberg has shown that there are highly significant correlations across cancer sites between the number of new drugs launched and changes in outcomes [42, 43]. Across countries, evidence further suggests an inverse relationship between the relative number of drugs available and mortality- or disability-related outcomes [44, 45]. When considering other factors that are likely to be confounders of the relationship between vintage and health outcomes (for example income and education), one study found that controlling for ten other potential determinants of longevity *increased* the estimated effect of pharmaceutical innovation by about 32% [46]. Given this literature, we assume a similar relationship to exist for adherence and drug vintage.

In parallel, marketing scholars consider consumer behavior models in which consumer perceptions and evaluations of product features influence repurchasing behavior [47, 48]. The attitudes towards perceived quality in product evaluations relative to other brands have been identified as strong predictors of brand satisfaction and loyalty which lead to product repurchase [49]. In the context of R&D-intensive markets, perceptions of brand innovativeness influence perceived product quality [47]. We argue that individuals who use higher quality goods are more likely to repurchase these goods compared to lower quality goods and that adherence reflects repurchase behavior. While the marketing literature has focused on identifying perceptions of product quality based on attitudinal evaluations of consumers, the economics literature has established the vintage concept that defines the relationship between product features and the overall product performance relative to other goods of the same type. Thus, the hypotheses that vintage is an indicator of product quality which implies that the average quality of newer (later-vintage) drugs is higher than the average quality of older (earlier- vintage) drugs, and that quality has a positive effect on adherence, imply that patients should be more adherent to newer drugs.

In considering the quality of the drug, we need to account for unobserved patient heterogeneity to perform a valid analysis of the effect of pharmaceutical innovation on adherence. Adherence behavior is influenced by socio-economic and demographic factors at the individual level [50, 51]. The healthy-adherer bias recognizes a tendency of individuals who otherwise engage in healthy behaviors to also be adherent to medication therapy. Such behaviors may be associated with higher socio-economic status [52]. In line with this, the marketing literature suggests that individual perceptions of product quality have been shown to differ systematically across consumer groups [53]. The setting in which a product is used is especially important if (1) the goods are experience goods and, (2) the consumer is, at least to some extent, a co-creator of the value gained in using a particular product (e.g. drug). Then, heterogeneity of the perceptions of product quality goes beyond demographic information [53, 54].

## Methods

### Data

We use the Truven Health Marketscan commercial claims and encounters database, which provides complete pharmacy and medical claims data for about 50 million insured employees and dependents between 2008 and 2013. For analysis, we use the 1 % random sample draw of enrollees provided by the National Bureau of Economic Research. To identify patients that initiate drug therapy and for whom adherence behavior could be measured for a period of 365 days, we included all individuals that were enrolled for more than three consecutive years.

Data on promotional activities (i.e. sampling) were obtained from the Medical Expenditures Panel Survey (MEPS), which includes data on prescription drug use by the US non-institutionalized population [55]. We measure marketing activities by the population-weighted number of free samples provided to patients at drug level on an annual basis [56]. At drug level, we then matched data on sampling at national level with our individual-level data to measure the extent of sampling of drugs that individual *i* uses, such that the level of sampling at drug class level varies across individuals by the specific drugs prescribed.

Drugs for chronic conditions were identified by National Drug Codes and the Truven Redbook [57]. We use the classification of a therapeutic class provided in the Redbook for the definition of drug classes. To measure drug vintage, we combined the prescription claims data with the National Drug Code Directory and the Drugs@FDA database [58].

### Cross sectional comparisons of adherence levels and drug vintage

We use cross-sectional comparisons to estimate the impact of drug vintage on adherence. Our unit of observation is drug class by patient. In observing patient-drug class combinations, we account for the fact that individuals with chronic conditions typically receive prescriptions from multiple drug classes that have different mechanisms and side effect profiles. We will account for unobserved heterogeneity of patients by using person-level fixed effects. For patient *i* receiving prescriptions in drug class *c*, we postulate the following general linear model:

1 $$ {adherence}_{ic}={\beta}_1\cdot RX\_{vintage}_{ic}+{\beta}_2\cdot {samples}_{ic}+\Pi \cdot {X}_{ic}+{\alpha}_{\mathrm{c}}+{\gamma}_i+{\varepsilon}_{ic}, $$

*adherence*_*ic*_ is the extent of individual *i*’s adherence to drugs in therapeutic class *c*, i.e. his or her medication possession ratio (MPR) during the first year of drug therapy. *RX*_*vintage*_*ic*_ denotes the mean vintage of drugs in therapeutic class *c* used by patient *i*, weighted by the number of days supplied of each drug in that class. *samples*_*ic*_ denotes the share of days supplied for which manufacturers have dispensed samples and which are used by individual *i*.^3^**Π*X***_***ic***_ is the vector of the covariates that influence adherence at drug class level. *γ*_*i*_ is a fixed effect for individual *i*. α_c_ is a fixed effect for therapeutic class *c*. *ε*_*ic*_ is the disturbance term.

We operationalize drug vintage (*RX*_*vintage*_*ic*_) by two variables, i.e., the Food and Drug Administration (FDA) approval year and the FDA therapeutic potential status. We hypothesize that adherence to newer (later-vintage) drugs is greater than adherence to older drugs. We use the year a drug was first approved by the FDA as our primary measure of drug vintage. This measure has been used previously to analyze the impact of pharmaceutical innovation on life expectancy and disability days [30]. Accordingly, the variable *FDA* _ *YEAR*_*ic*_ denotes the weighted mean FDA approval year of the drugs in class *c* consumed by patient *i*, weighted by the number of days supplied to individual *i* of each drug in that therapeutic class.^4^ If a patient received multiple active ingredients in the same drug class, we weight the FDA approval year by the number of days supplied of drugs approved in that year. We further classify the year of FDA approval into four periods: before 1980, between 1981 and 1990, between 1991 and 2000, and after 2000.

FDA drug approval requires that a drug be safe and effective, but not that it be superior in comparison with other drugs [59]. When the FDA begins to review a new drug for approval, it assesses the drug’s “therapeutic potential,” i.e. whether it is an advance over available therapies [60]. Accordingly, the FDA designates the drug as either a priority-review drug or as a standard-review drug. Besides year of drug approval, we consider this classification as an indicator of a drug’s *effective vintage* compared to its *actual* vintage [30]: the effective vintage of a standard-review drug may be earlier than its actual vintage.^5^ We define the variable *priority*%_*ic*_ as the share of days supplied to individual *i* of drugs in drug class *c* that have been awarded priority review status.^6^

We control for situational factors of adherence behavior expressed by the term ***X***_***ic***_ of eq. (1) which includes both the pecuniary and non-pecuniary costs of adhering to drug therapy. We measure promotional activities by the number of free samples provided at the national level in a drug class, defined by the variable *samples*. Controlling for promotional activity could cause the effect of vintage on adherence to be underestimated, since it has been shown that newer drugs are more frequently advertised [61]. As promotional activity typically increases utilization of competing drugs and that of the promoted brand [62], we do not account for variations in promotional activity at brand level. Direct pecuniary cost is typically expressed by the level of copayments for which we calculate the patient copayment per day of therapy (variable *copay* _ *day*). Other non-pecuniary cost refers to factors other than out-of-pocket payments that may enhance or hinder medication adherence. We included the share of prescriptions filled via mail-order pharmacy (variable *mail*%). Finally, we control for the year in which an individual started drug therapy.

### Accounting for unobserved heterogeneity of patients

It has been shown that there are likely to be both observed (e.g. socio-economic status) and unobserved (e.g. “healthy-adherer”) individual characteristics that influence adherence behavior [52, 63]. The healthy-adherer effect has been documented widely in the literature [52, 64–66]. It arises when patients who adhere to one type of therapy or prevention are more likely to engage in other healthy behaviors than their non-adherent counterparts, independent of which type of technology they use. In line with this, the literature on the effect of adherence on health outcomes has documented that not accounting for unobserved heterogeneity of healthy adherer behaviors can result in biased estimates of the effect of adherence on clinical outcomes when adherence is considered as exogenous variable [52, 64, 67]. Steiner [66] argues that single sociodemographic and clinical characteristics to guide adherence cannot predict adherence because each individual carries out a set of behaviors that interact with each other and are influenced by social and environmental considerations as well as clinical care. Belotti et al. document that the returns to adherence on health are highly heterogeneous by treatment regime and physicians [63]. Moreover, heterogeneity of patients and physicians is an important determinant of health and moderates the effect of adherence on health. Besides, estimates of adherence on health are upward biased and, the moderation effect of patient heterogeneity on adherence is larger than physician heterogeneity. Accordingly, we assume that accounting for unobserved patient heterogeneity is equally important when adherence is considered as endogenous variable.

In this study, we observe adherence to different types of drugs used by the same individual but not other health-related behaviors such as prevention. We include patient level fixed effects, denoted by *γ*_*i*_ for individual *i* to account for characteristics (e.g. age, education, marital status, number of comorbidities) that are invariant across a patient’s medical condition and may be correlated with drug vintage, including healthy-adherer behaviors. Moreover, we assume that physician-patient interactions are captured in the patient fixed effects, especially preferences of the providers visited and health plan characteristics at individual level. Choices made by prescribers and pharmacies for the underlying drug formularies such as prior authorization or other choices and the interactions with patient behavior likely influence patient behavior [68]. Of note is that patient level fixed effects do not capture whether the same individual behaves differentially across different technologies (i.e. drugs) used that relate to the within individual level heterogeneity of environment, socio-economic factors, tastes and preferences besides the quality of the drug.

Descriptive analysis of our sample data shows that the number of patients using multiple drug classes is substantial. 87% of patients are prescribed at least two drug classes or more in the first year of treatment when conditioning on a patient having a diagnosis for a chronic disease as described below. For example, our sample includes data on 10,741 hypertension patients. The mean number of drug classes per patient is 5.63, so the number of observations is 10,741*5.63 = 60,525 observations. To analyze the extent to which, conditional on being a below- (above-) average adherer to one class of drugs, the individual is also a below- (above-) average adherer to other classes, we calculated bivariate correlations between adherence levels of the same individual to all drug classes considered in the empirical analysis at the drug class level. Using an unbalanced correlation matrix, we find that across 128 drug classes, the median correlation is 0.13. Calculating Pearson correlations of the Medicines Possession Ratio within patients, we reject the null hypothesis that these correlations are equal to zero. Table 1 shows a matrix of the unbalanced cross correlations of the 10 drugs classes most frequently used by patients that are conditional on the patients receiving prescriptions in both drug classes.

**Table 1:** Pearson Correlation Coefficients of Medicines Possession Ratio of Patients across different Therapeutic Classes

	*051 Cardiac Beta Blockers*	*027 Sympathomimetic Agents NEC*	*138 Antiinflam Agents EENT NEC*	*047 Cardiac ACE Inhibitors*	*168 Contraceptive Oral Comb*	*162 Gastrointestinal Drugs*	*053 Antihyperlipidemic Drugs*	*166 Adrenals Comb NEC*	*059 Analg Antipyr Nonsteroid*	*069 Psychother Antidepressants*
*051 Cardiac Beta Blockers*	1 (5939)	0.069 (405)	0.153** (539)	0.379*** (1805)	0.183** (241)	0.319*** (1240)	0.413*** (2375)	0.105*** (982)	0.201*** (1208)	0.31157*** (1445)
*027 Sympathomimetic Agents NEC*		1 (6196)	0.244*** (1071)	0.189*** (496)	0.047 (417)	0.175*** (877)	0.117** (731)	0.371*** (2708)	0.116*** (803)	0.15459*** (1044)
*138 Antiinflam Agents EENT NEC*			1 (7283)	0.201*** (748)	0.189*** (565)	0.282*** (1165)	0.233*** (1126)	0.310*** (1737)	0.235*** (1139)	0.19879*** (1374)
*047 Cardiac ACE Inhibitors*				1 (7780)	0.261** (177)	0.277*** (1323)	0.432*** (3120)	0.152*** (1088)	0.200*** (1528)	0.310*** (1573)
*168 Contraceptive Oral Comb*					1 (7915)	0.189*** (583)	0.231*** (222)	0.103** (833)	0.178*** (914)	0.193*** (1548)
*162 Gastrointestinal Drugs*						1 (8822)	0.343*** (2134)	0.226*** (1821)	0.276*** (1873)	0.325*** (2297)
*053 Antihyperlipidemic Drugs*							1 (10,964)	0.150*** (1646)	0.178*** (2171)	0.308*** (2379)
*166 Adrenals Comb NEC*								1 (11,080)	0.178*** (2101)	0.134*** (2197)
*059 Analg Antipyr Nonsteroid*									1 (11,909)	0.225*** (2641)
*069 Psychother Antidepressants*										1 (13,298)

Notes: ***: *p* < 0.0001; **: *p* < 0.01, number of observations in parantheses. Source: Truven Health Marketscan Commercial Claims and Encounters, 2008–2013, 1% random sample as provided by National Bureau of Economic Research

To assess the appropriateness of person-level fixed effects empirically, we also estimate a set of regression models including the patient’s age, gender and information about comorbidity structure instead (Supplementary Material). Chi-squared statistics suggested the superiority of the fixed effects approach.^7^ In the models that include individual level fixed effects, F-statistics suggest that individual level fixed effects explain a significant portion of the variance.

### Measures of medication adherence

We measure adherence levels by the medication possession ratio (MPR) in the 365 days after the first prescription for patient *i* in drug class *c*. The MPR is the days supplied of the drug class dispensed during the follow-up year divided by the number of days in that same year. We truncated MPRs higher than 100% because we cannot distinguish inappropriate behaviors such as overuse and early refills from appropriate behaviors such as changes in drug regimens or combination therapies [4]. Within the same drug class, physicians may switch therapy within the class for different reasons. This is the case in about 15% of patient/drug-class observations of our sample. In that case, we calculated weighted averages for our outcome measures and the by the number of days supplied by the physician.^8^ Also, we calculated the weighted average of FDA approval year by the number of days supplied and adherence levels in the same period of time. Thus, for our variable of interest, we measure whether on average the mean vintage of drugs in a particular drug class is related to improved adherence behavior, i.e. as in the footnote $RX\_{vintage}_1\cdot \frac{120}{365}+R{X_{vintage}}_2\cdot \frac{245}{365}=2003.08$.

### Identification of patients and health conditions

We analyze adherence levels of patients in their first year of drug therapy since adherence levels vary by the duration of drug therapy [69]. Thus, we restrict our sample to patients who were considered healthy and initiate medical encounters for the first time to control for initial health status. We include pharmacy claims of individuals if they showed no pharmacy, inpatient or outpatient claims in a period of at least twelve months before the indexing date, the earliest possible date as of January 1st 2008. We use this approach also to reduce potential reverse causality issues.

We use two different strategies to identify start of drug therapy. First, we identify the initiation of drug therapy by the earliest date a patient received prescriptions in drug class *c* (models 1–2). As a second method, we identify patients by their earliest date of diagnosis recorded in the claims data in a set of five chronic conditions (models 3–7). Based on ICD-9-CM classifications, we include hypercholesterolemia, hypertension, diabetes, hypothyroidism and, osteoporosis, as they are common in the US employed population [4]. Patients needed to have at least two claims for the same diagnosis to reduce the risk that diagnoses were not stated randomly. In the disease-specific regressions (models 3–7), the aim was to obtain condition-specific estimates of the effect of drug vintage on adherence. Here, we exclude drug classes with a prescription volume of less than 2 % per condition. The Chow test indicates that it is appropriate to estimate five separate regression models by chronic condition [70]. All statistical analyzes were performed using SAS version 9.4.

## Results

### Descriptive analysis

Table 2 shows descriptive statistics of our data.^9^ The mean FDA approval year of the drugs studied in the full sample is 1982 with a standard deviation of 16 years, indicating large variation in the vintages of drugs used. Most prescriptions filled during the period 2009–2013 (39%) are for drugs approved between 1991 and 2000. Across the full sample, 43% of days supplied are for drugs that had FDA priority-review status. 18% of days supplied are filled via mail-order pharmacies. The average copayment per day is $0.47.

**Table 2:** Mean medicines possession ratio and FDA approval year of top 30 therapeutic drug classes

*Therapeutic drug class*	*N (patients)*	*MPR: mean (std)*	*FDA approval year: mean (std)*
*069-Psychother Antidepressants*	17,683	58.49% (31.36%)	1990.64 (10.39)
*059-Analg/Antipyr Nonsteroid/Antiinflam*	15,794	21.11% (22.85%)	1983.02 (10.22)
*053-Antihyperlipidemic Drugs NEC*	14,841	67.12% (30.07%)	1993.21 (7.01)
*166-Adrenals & Comb NEC*	14,471	16.92% (22.32%)	1968.29 (16.18)
*162-Gastrointestinal Drugs Misc NEC*	12,020	51.95% (32.23%)	1994.03 (5.84)
*168-Contraceptive Oral Comb NEC*	10,929	67.88% (30.97%)	1958.05 (5.59)
*047-Cardiac ACE Inhibitors*	10,702	69.44% (30.37%)	1986.04 (2.93)
*138-Antiinflam Agents EENT NEC*	9429	29.83% (20.32%)	1992.80 (4.54)
*051-Cardiac Beta Blockers*	8293	68.36% (30.99%)	1985.14 (10.35)
*027-Sympathomimetic Agents NEC*	8133	19.01% (16.63%)	1983.34 (4.57)
*178-Thy/Antithy Thyroid Hormones*	6579	78.60% (26.73%)	1999.55 (3.91)
*052-Cardiac Calcium Channel*	6037	71.15% (30.17%)	1989.76 (4.74)
*174-Antidiabetic Agents Misc*	5496	64.46% (29.56%)	1996.22 (2.78)
*046-Cardiac Drugs. NEC*	5467	73.42% (28.65%)	1990.78 (8.38)
*068-Anticonvulsants Misc*	5443	53.69% (32.01%)	1992.72 (6.23)
*170-Estrogens & Comb NEC*	4693	60.43% (32.18%)	1972.23 (13.94)
*071-Stimulant Amphetamine Type*	4651	57.29% (29.00%)	1973.63 (21.11)
*248-Leukotriene Modifiers*	4515	53.25% (30.72%)	1997.99 (0.16)
*224-Vitamin D NEC*	4490	37.21% (24.96%)	1942.43 (7.41)
*124-Diuretics Thiazides & related*	4435	66.21% (31.34%)	1959.60 (3.24)
*195-Antiinflam S/MM Agnts & Comb Misc*	3415	12.50% (10.25%)	1963.85 (12.05)
*234-Unclassified Agents NEC*	3303	58.29% (32.92%)	1996.60 (5.79)
*001-Antihistamines & Comb NEC*	3044	42.70% (29.41%)	2002.64 (7.67)
*177-Progestins NEC*	2606	47.57% (33.57%)	1962.49 (4.97)
*123-Diuretics Potassium-Sparing*	2591	64.39% (30.79%)	1964.61 (4.38)
*011-Antibiot Tetracyclines*	2550	36.08% (23.85%)	1968.61 (5.38)
*250-Phosphodiesterase Inhibitors*	2416	25.46% (23.80%)	2000.58 (2.45)
*232-Multivit Prep Multivit Prenatal*	2377	52.82% (26.91%)	1967.65 (4.02)
*060-Anal/Antipyr Opiate Agonists*	2288	28.06% (33.40%)	1966.08 (18.82)

Source: Truven Health Marketscan Commercial Claims and Encounters, 2008–2013, 1% random sample as provided by National Bureau of Economic Research

We identify considerable variation in vintage at drug class level by chronic condition in our study sample. Table 3 shows the mean MPR and FDA approval year for the 30 drug classes with the highest prescription volume.

**Table 3:** Descriptive Statistics, start of therapy defined by first time therapeutic class has been prescribed

*Characteristic (variable name)*	*Unit*	*Full sample*
*Sample size (patient - drug class combinations)*	*n*	247,146
*Sample size (patients)*	*n*	70,896
*Adherence (MPR)*	*mean (STD)*	49.99% (34.13%)
*FDA approval year (FDA_year)*	*mean (STD)*	1982.32 (16.48)
*% days supplied with FDA approval year*		
*before 1980*	*%*	31.06%
*between 1981 and 1990*	*%*	20.74%
*between 1991 and 2000*	*%*	39.14%
*after 2000*	*%*	9.06%
*FDA priority status (priority%)*	*%*	42.84%
*Promotional activity (number of free samples received by patients in drug class for each 100 days supplied per 1,000,000 US non-institutionalized population)*	*mean (STD)*	1.11 (3.26)
*Mail-order prescriptions (mail%)*	*%*	17.87%
*Copayment / day (copay_day)*	*mean (STD)*	0.47 (0.97)
*Patient age (age)*	*mean (STD)*	42.60 (0.49)
*Patient gender (% male)*	%	40.11%

FDA: Food and Drug Administration; Sources: Truven Health Marketscan Commercial Claims and Encounters, 2008–2013, 1% random sample as provided by National Bureau of Economic Research; Drugs@FDA Data Files (https://www.fda.gov/drugs/drug-approvals-and-databases/drugsfda-data-files)

### Effect of vintage on adherence in first year of drug therapy by therapeutic class

Estimates of eq. (1) when the five chronic conditions are pooled are presented in Table 4. Our results suggest that adherence increases (i.e., the MPR is higher) if the drug used in a particular drug class is newer and if it has received priority review status. In Model 1, the MPR increases by 0.0025 if the FDA approval year increases by one year. This means that a 10-year increase in the FDA approval year raises the mean MPR by 2.5 percentage points across all therapeutic classes, on average. If the drug has received FDA priority review status, the MPR increases by 0.0087. Moreover, we find a small positive significant effect of the number of samples provided on the MPR of 0.0019. This means that when one additional sample was provided per 100 days supplied in 1 million population, the MPR increases by 0.19 percentage points. This further suggests a small indirect effect of innovation through promotional activities. If the share of prescriptions filled by mail-order pharmacy increases by one percentage point, the MPR increases by 3.29 percentage points (*p* < 0.001). A higher copayment per day has a negative effect on adherence levels (−0.0362 if copayment per day increased by one dollar, p < 0.001).

**Table 4:** Estimates of medicines possession ratio by product vintage – pooled regressions

		*Model 1 (drug vintage continous)*	*Model 2 (drug vintage by decades)*	*Model 1a (no person level FE)*	*Model 1b (excluding situational factors)*
**Drug quality**					
*FDA approval year (FDA_year)*		0.0025*** (0.0001)		0.0030*** (0.0001)	0.0026*** (0.0001)
*FDA approval year (categorical)*	*later than 2000*		0.0804*** (0.0035)		
	*between 1991 and 2000*		0.0821*** (0.0026)		
	*between 1981 and 1990*		0.0424*** (0.0025)		
	*before 1980*				
*FDA priority status (priority%)*		0.0087*** (0.0019)	0.0128*** (0.0020)	0.0090*** (0.0018)	0.0112*** (0.0020)
***Situational factors***					
*Promotional activity (samples)*		0.0019*** (0.0000)	0.0021*** (0.0000)	0.0023*** (0.0000)	
*Share of days supplied* via *mail-order pharmacy (mail%)*		0.3289*** (0.0035)	0.3302*** (0.0035)	0.1876*** (0.0016)	
*Prices: copayment / day (copay_day)*		−0.0362*** (0.0009)	−0.0351*** (0.0009)	−0.0376*** (0.0008)	
*Fixed effect individual*		yes	yes	no	yes
*Fixed effect therapeutic class*		yes	yes	yes	yes
*Fixed effect year started therapy*		yes	yes	yes	yes
*R-Squared*		0.717	0.717	0.391	0.695
*Root MSE*		0.218	0.218	0.267	0.226
N		248,597	248,597	248,597	248,597

Note: ***:p < 0.0001; **:p < 0.01; * < 0.05; FDA: Food and Drug Administration; Standard errors are in parantheses. Sources: Truven Health Marketscan Commercial Claims and Encounters, 2008–2013, 1% random sample as provided by National Bureau of Economic Research; Drugs@FDA Data Files (*http://www.fda.gov/drugs/drug-approvals-and-databases/drugsfda-data-files**)*

Model 2 categorizes the FDA approval year into four periods. Compared to drugs that have been approved before 1980, the effect of drug vintage on MPR is highest for drugs approved for the period between 1991 and 2000 (estimate: 0.0821) and lowest for the period between 1981 and 1990 (estimate: 0.0424). Apart from this, the effect sizes and significance levels of the covariates that reflect situational factors are similar in size and direction compared to Model 1.

When we completely refrain from potential unobserved heterogeneity at individual level and exclude individual fixed effects as expressed by *γ*_*i*_, the estimate of FDA approval year increases slightly to 0.003 (Model 1a). In that case, the effect of vintage is slightly upward biased. Second, the effect of vintage on adherence increases to 0.0026 when we exclude price, promotion and distribution channel.

### Effect of vintage on adherence in first year of drug therapy in five chronic conditions

Table 5 displays regressions (models 2–7) that stratify individuals by five chronic conditions. Again, we find significant effects of drug vintage on medication adherence in all chronic conditions analyzed, with the size of the effect of FDA approval year somewhat varying across conditions. The effect of FDA approval year on MPR is highest in hypothyroidism and osteoporosis (estimate: 0.0036) and lowest in diabetes (estimate: 0.0023). Whether a drug has received FDA priority-review status has significant positive effects in the models of hypertension, hypothyroidism and osteoporosis. Promotional activities in terms of sampling significantly influenced adherence levels in the models of hypertension, hypercholesterolemia and osteoporosis. Effect sizes and significance levels of the other variables that influence adherence are similar compared to the results of the pooled regressions (models 1 and 2). Estimates of the effect of vintage on adherence are much smaller when we included person level fixed effects that relate to both observed and unobserved heterogeneity instead of including patient characteristics and comorbidity only that captures observed heterogeneity. For example, the estimate of FDA approval year decreased to 0.0029 compared to 0.0053 in hypertension patients.

**Table 5:** Estimates of medicines possession ratio by product vintage – regressions by five major health conditions

	*Hypertension (Model 3)*	*Hyper-cholesterolemia (Model 4)*	*Diabetes (Model 5)*	*Hypothyroidism (Model 6)*	*Osteoporosis (Model 7)*
**Drug quality**					
*FDA approval year (FDA_year)*	0.0029*** (0.0003)	0.0031*** (0.0003)	0.0023*** (0.0005)	0.0036*** (0.0005)	0.0036*** (0.0006)
*FDA priority status (priority%)*	0.0262*** (0.0060)	0.0080 (0.0064)	0.0183 (0.0096)	0.0323* (0.0128)	0.0321* (0.0149)
***Situational factors***					
*Promotional activity (samples)*	0.0033** (0.0010)	0.0037** (0.0010)	0.0028 (0.0020)	0.0065** (0.0020)	0.0030 (0.0020)
*Share mail-order prescriptions (mail%)*	0.3579*** (0.0134)	0.3282*** (0.0137)	0.3367*** (0.0220)	0.3373*** (0.0297)	0.3850*** (0.0327)
*Prices: copayment / day (copay_day)*	−0.0294*** (0.0042)	−0.0350*** (0.0046)	−0.0644*** (0.0085)	−0.0091 (0.0064)	−0.0284* (0.0125)
*Person-level Fixed Effects*	yes	yes	yes	yes	yes
*Therapeutic Class Fixed Effects*	yes	yes	yes	yes	yes
*Year started therapy*	yes	yes	yes	yes	yes
*Patient - drug class combinations (N)*	60,525	49,431	23,249	16,778	9757
*Patients (N)*	10,741	10,061	3455	3232	1981
*Therapeutic Classes (N)*	13	13	16	12	12
RSquare	0.760	0.803	0.774	0.795	0.795
RootMSE	0.195	0.182	0.186	0.187	0.197

Note: ***:p < 0.0001; **:p < 0.01; * < 0.05; FDA: Food and Drug Administration; Standard errors are in parantheses. Sources: Truven Health Marketscan Commercial Claims and Encounters, 2008–2013, 1% random sample as provided by National Bureau of Economic Research; Drugs@FDA Data Files (*https://www.fda.gov/drugs/drug-approvals-and-databases/drugsfda-data-files**)*

**Fig. 1:**
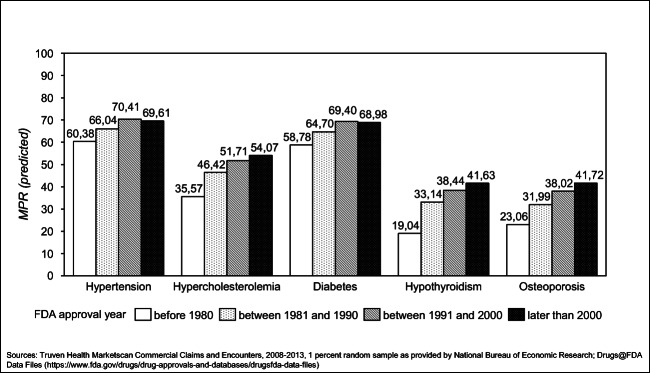
Predicted effect of drug vintage on medicines possession ratio (MPR) by FDA approval year in patients in their first year of therapy, US employed population, controlling for situational factors (promotional activity, copayments and distribution channel), including individual level and drug class-level fixed effects

Figure 1 shows estimates of mean MPR by vintage category for each of the chronic conditions (models 3–7). We observe a strong increase in mean MPR when we compare drugs approved before 1981 with drugs approved after 1981 or after 1991. For drugs approved in the period after the year 2000, adherence levels are still higher compared to drugs before 1980. However, the increase is smaller or slightly lower compared to drugs approved in the period between 1991 and 2000. For hypertension and diabetes, adherence levels decrease slightly, but the 1991–2000 vs. post-2000 differences are not statistically significant.

### Robustness

Several robustness analyses substantiated the results (Supplementary Material). We a) accounted for the distributional nature of the outcome variable using log-odds of MPR instead of natural units, b) considered patient subgroups that may be more or less vulnerable to medication non-adherence (e.g. patients that do not switch across drugs in the same class, patients that use multiple brands, c) tested the mediating effects of copayment and promotional activity. Especially, part of the effect of co-payments seems to be attenuated by the level of vintage as newer drugs tend to have higher co-payments. When we exclude the variable that controls for the level of co-payment, the effect of adherence decreases in all model specifications suggesting trade-offs between the level of innovation and copayments. Finally, our results are robust when estimating models using aggregate longitudinal data. Across all robustness analyses, the effect of vintage on medication adherence was consistently significant, although somewhat varying in magnitude of the effect.

## Discussion

This study has investigated the relationship between drug quality that we captured by a drug’s vintage as measure of productivity of treatment and medication adherence in the US working population. We demonstrate that patients tend to be more adherent to newer drugs. The medicine possession ratio increases by 2.5 percentage points when the mean vintage of a drug increases by ten years. This effect may appear small, but our estimates indicate that a 10-year increase in drug vintage has the same effect on medication adherence as a 0.35 USD reduction in copayment per day of therapy. Also, our estimates of vintage reflect averaged effects across all drugs of the same vintage, some of which may be more effective in terms of effectiveness than others [39, 40].

The effect of vintage on adherence is similar in magnitude to the effects of copayments and larger than that of promotional activity [71, 72]. A one standard deviation increase in FDA approval year is associated with an increase in adherence of 4.12 percentage points, while a one standard deviation reduction in copayment per day is associated with an increase in adherence of 3.51 percentage points. A one standard deviation increase in promotional activity is associated with an increase of 0.61 percentage points. While drugs certainly differ in efficacy for different types of condition, different levels of severity, co-morbidity with other conditions, physicians and pharmacy benefit managers should be aware that, on average, patients that use newer drugs are more adherent compared to using older drugs when making choices about certain drug agents or formulary design.

Our results further support the notion that promotional activity – measured by sampling activities of drug companies at national level – has a very small effect on adherence levels. This result confirms previous findings regarding changes in overall therapy initiation conditional on marketing activity [72]. However, effect sizes are small or economically not relevant in influencing adherence levels [73, 74] and this result is likely to be independent from the measure of promotional activity, as detailing and sampling were shown to be complementary [75]. Most studies that have analyzed influences from economic incentives on adherence have focused on variations in co-payment levels [76]. Our results suggest that part of the effect of co-payments may be attenuated by the level of innovation as newer drugs tend to have higher co-payments.

In this study, we demonstrate that biomedical innovation may not only have direct effects on health outcomes and health care consumption but also indirect effects via adherence. Existing studies show significant effects of drug vintage on mortality and expenditures [43–45, 77]. We contribute to this literature by showing that biomedical innovation influences adherence, and numerous studies have shown that adherence influences health outcomes. Adherence has been identified as one of the most important factors in explaining health outcomes [63, 78]. We show that adherence levels are positively influenced by the level of drug quality (expressed by vintage) such that adherence is an important mechanism behind the relationships between drug vintage and mortality and expenditure. Thus, part of the effects of biomedical innovation on health outcomes can be attributed to the indirect effect of adherence on outcomes. Besides, we confirm the importance of accounting for patient heterogeneity to explain variations beyond sociodemographic and comorbidity characteristics in adherence levels when adherence is endogenous [66]. Estimates of adherence on health that do not account for the moderating effects of biomedical innovation and patient heterogeneity on adherence may thus lead to biased estimates of the influence of biomedical innovation on health. Managing improvements in adherence levels can be addressed more directly by health care providers than long-term effects, as these are often influenced by a large number of factors.

The magnitude of the effect on adherence of using later vintage drugs is similar to, or smaller than, the effect of some interventions. Two studies found that the percentage of individuals being adherent at a level higher than 80% increased by about 3.5 to 4.7 percentage points conditional on copayment structures of health care plans [17, 21]. While we aimed to reduce biases on adherence levels by analyzing individuals in their first year of drug therapy and with a diagnosis of a chronic condition, we cannot observe the explicit prescription decisions of using later vintage drugs compared to earlier vintage drugs. Additionally, our estimates of the effects on adherence of variables that account for the non-pecuniary and pecuniary costs are similar to previous findings despite our focus on the privately-insured employed population [18, 19, 52].

We need to mention some limitations. Using claims data, we are unable to identify individuals who never filled a prescription (“primary non-adherence”). Second, we cannot observe whether patients actually consumed the drugs prescribed. Often, mail-order prescriptions are re-filled automatically. Another limitation is that we could not incorporate information about adverse events and side effects, another indicator of drug quality that may reduce adherence. Prescribing behavior is adjusted when new information about adverse events becomes available [79]. Fourth, we cannot account for heterogeneity that may arise in the same patient across different drug classes besides drug vintage, although the evidence on the healthy adherer bias suggests that patient level heterogeneity seems most important to account for individual preferences, comfort and experience. However, if individuals are differentially adherent to different drugs because of within-individual variation in factors such as preferences, comfort and experience, this could cause a potential source of endogeneity in our analysis. Relevant aspects are dimensions of side effects. For example, suppose that a patient is male and attempting to conceive a child with his partner. This patient may adhere perfectly well to drug classes that have no sexual side effects, but be a poor adherer to drugs that do. Previous approaches have emphasized individual level heterogeneity that relates to the healthy adherer bias. Future research may aim to explicitly capture within individual level heterogeneity across different drug classes. Finally, we cannot control for the mix of providers that the patient is using. In our sample, 15% of patients have received services from a single provider in the observation period. Provider decisions might contribute to variation in medication adherence and may differ in the vintage of drugs they prescribe [80, 81].

## Conclusions

Our study analyzes the influence of drug vintage on medication adherence in the US employed population. We show that patients are more adherent to newer drugs within a therapeutic class, controlling for unobserved individual effects and situational factors. Patients are also more adherent to priority-review drugs, which are of later “effective vintage” than standard-review drugs. We identify promotional activities as an additional channel through which drug quality may influence adherence. Prescribers and pharmacy benefit managers should be aware that patients tend to be more adherent to newer drugs within a therapeutic class when making prescription choices.

## Electronic supplementary material

ESM 1 (PDF 370 kb)

## Footnotes

1 We consider quality in terms of the effectiveness of a drug which comprises all features (including clinical effectiveness and side-effect profiles) to affect a treatment episode. It is not restricted to the quality of the pharmaceutical product that stems from the manufacturing process.

2 One definition of vintage is “a period of origin or manufacture.” [37]

3 *samples*_*ic*_ = (∑_*d*_*DAYSUPP*_*idc*_ ∙ *samples*_*d*_)/(∑_*d*_*DAYSUPP*_*idc*_), where *DAYSUPP*_*idc*_ is the number of days of drug *d* in therapeutic class *c* supplied to individual *i*, and *samples*_*d*_ is number of samples reported in the Medical Expenditures Panel Survey in the year the patient used drug *d*.

4 *FDA* _ *YEAR*_*ic*_ = (∑_*d*_*DAYSUPP*_*idc*_ ∙ *FDA* _ *YEAR*_*d*_)/(∑_*d*_*DAYSUPP*_*idc*_), where *DAYSUPP*_*idc*_ is the number of days of drug *d* in therapeutic class *c* supplied to individual *i*, and *FDA* _ *YEAR*_*d*_ is the FDA approval year of drug *d*.

5 For example, if a standard review drug approved in 2013 is considered equivalent to a drug approved in 2003, its effective vintage is 2003 whereas its actual vintage is 2013.

6 *PRIORITY*%_*ic*_ = (∑_*d*_*DAYSUPP*_*idc*_ ∙ *PRIORITY*_*d*_)/(∑_*d*_*DAYSUPP*_*idc*_), where *PRIORITY*_*d*_ = 1 if any product containing drug *d* was priority review and 0 otherwise.

7 Using the absorb statement of the GLM procedure in SAS, we also include patients using one drug class only. Results were similar with regard to effect sizes and significance levels when we excluded these patients.

8 For example, a patient starts simvastatin, which obtained FDA approval in 1991, and her LDL (bad cholesterol) does not decline sufficiently after six months. For example, this therapy lasts 245 days for which the patient is not fully adherent because the number of days supplied was 200, i.e. ${MPR}_1=\frac{200}{245}=81.63\%$. The physician then switches to a newer statin that the patient remains on for the remainder of the year which also increased HDL (good cholesterol) levels, for example pitavastatin that was approved in 2009. Again, we capture the number of days supplied, e.g. 120 days or ${MPR}_2=\frac{120}{120}=100\%$ such that the weighted MPR in the statin class is ${MPR}_1\ast \frac{245}{365}+{MPR}_2\ast \frac{120}{365}=87.67\%$.

9 Descriptive statistics for patients identified by health condition are documented in the Appendix Tables.
