# Supplementary material for: Are patients more adherent to newer drugs?
Source: Health Care Manag Sci. 2020 Aug 8;23(4):605–18. doi: 10.1007/s10729-020-09513-5 (PMC7674371; doi:10.1007/s10729-020-09513-5)
Supplement: Supplementary file 1 — (PDF 370 kb) [file 10729_2020_9513_MOESM1_ESM.pdf]

Are patients more adherent to newer drugs?

Supplementary Material

Supplementary Material 1 – Regressions using patient characteristics to account for unobserved heterogeneity

In contrast to regressions that use person fixed effects, the model is now defined as $adherence_{ic} = \beta_1 RX\_vintage_{ic} + \mathbf{\Pi X_{ic}} + \boldsymbol{\gamma} \mathbf{Z_{ic}} + \alpha_c + \varepsilon_{ic}$. $\boldsymbol{\gamma}\mathbf{Z_{ic}}$ is a vector that includes the variables patient age, patient gender and dummy variables to describe the patient's comorbidity structure, and, ε_{ic} is the disturbance term. We used the H-CUP Chronic Condition Indicator[1] to specify dummy variables by body system assignments to adjust for comorbidity structure.

	Hypertension (Model 3)	*Hyper-cholesterolemia (Model 4)*	*Diabetes (Model 5)*	*Hypothyroidism (Model 6)*	*Osteoporosis (Model 7)*
FDA approval year (FDA_year)	0.0053***	0.0053***	0.0029***	0.0055***	0.0042***
	(0.0002)	(0.0002)	(0.0003)	(0.0003)	(0.0003)
FDA priority status (priority%)	0.0471***	0.0389***	0.0500***	0.0332***	0.0234**
	(0.0037)	(0.0036)	(0.0063)	(0.0072)	(0.0078)
Promotional activity (samples)	0.0006***	0.0007***	0.0006***	0.0008***	0.0008***
	(0.0001)	(0.0001)	(0.0001)	(0.0001)	(0.0001)
Share mail-order prescriptions (mail%)	0.1312***	0.1306***	0.1308***	0.1335***	0.1478***
	(0.0030)	(0.0031)	(0.0051)	(0.0055)	(0.0080)
Copayment / day (copay_day)	-0.0623***	-0.0591***	-0.0621***	-0.0442***	-0.0504***
	(0.0026)	(0.0027)	(0.0047)	(0.0040)	(0.0044)
Patient Age	0.0038***	0.0031***	0.0041***	0.0017***	0.0026***
	(0.0001)	(0.0001)	(0.0002)	(0.0002)	(0.0003)
Patient Gender (ref: female)	0.0206***	0.0246***	0.0184***	0.0195**	-0.0058
	(0.0024)	(0.0026)	(0.0043)	(0.0057)	(0.0086)
HCUP comorbidy indicators	✓	✓	✓	✓	✓
Therapeutic Class Fixed Effects	✓	✓	✓	✓	✓
Year started therapy	✓	✓	✓	✓	✓
RSquare	0.4451	0.5029	0.4770	0.5554	0.5326
RootMSE	0.2680	0.2573	0.2585	0.2444	0.2636
N (patient - drug class combinations)	53,100	43,280	16,993	13,964	8,116

[1] HCUP (2016) Chronic Condition Indicator (CCI) for ICD-9-CM. Available at: https://www.hcup-us.ahrq.gov/toolssoftware/chronic/chronic.jsp#files (Accessed: 25 July 2019).

SUPPLEMENTARY MATERIAL 2 – DESCRIPTIVE STATISTICS BY MAJOR HEALTH CONDITIONS

Characteristic (variable name)	*Unit*	*Full Sample*	*Hypertension*	*Hyperchol*	*Diabetes*	*Hypothyroidism*	*Osteoporosis*
Sample size (patient - drug class combinations)	*n*	159,740	60,525	60,525	23,249	9,757	16,778
Sample size (patients)	*n*	29,835	10,856	10,199	3,497	3,278	2,005
Adherence (MPR)	*mean*	67.67%	67.42%	67.65%	68.92%	69.86%	62.61%
	(STD)	(38.07%)	(37.74%)	(38.45%)	(37.12%)	(38.00%)	(39.99%)
FDA approval year (FDA_year)	*mean*	1987.50	1985.70	1988.52	1988.91	1990.02	1986.61
	(STD)	(12.38)	(12.46)	(11.58)	(11.08)	(13.61)	(14.40)
% days supplied with FDA approval year							
- before 1981	*%*	12.68%	13.70%	8.39%	12.91%	18.65%	13.35%
- between 1981 and 1990	*%*	25.46%	28.70%	25.69%	23.02%	18.95%	18.57%
- between 1991 and 2000	*%*	52.90%	48.08%	55.76%	42.78%	56.94%	61.71%
- after 2000	*%*	8.95%	9.19%	9.53%	11.58%	4.14%	6.08%
FDA priority status (priority%)	*%*	36.58%	38.90%	34.03%	42.02%	26.47%	40.80%
Marketing (samples)	*mean*	0.86	0.78	1.02	0.72	0.74	1.07
	(STD)	(2.27)	(2.17)	(2.45)	(1.96)	(2.16)	(2.75)
Mail-order prescriptions (mail%)	*%*	23.35%	22.29%	25.34%	23.73%	22.06%	21.14%
Copayment / day (copay_day)	*mean*	0.42 (0.56)	0.39 (0.53)	0.43 (0.55)	0.44 (0.57)	0.39 (0.56)	0.47 (0.72)

FDA: Food and Drug Administration

SUPPLEMENTARY MATERIAL 3 – ESTIMATES OF MEDICINES POSSESSION RATIO EXCLUDING PROMOTIONAL ACTIVITY

a) pooled regressions

		Model 1 (drug vintage continous)			Model 2 (drug vintage by decades)		
Intercept							
FDA approval year (FDA_year)		0.0027	0.000	***			
FDA approval year (categorical)	*later than 2000*				0.0943	0.0033	***
	between 1991 and 2000				0.0835	0.0025	***
	between 1981 and 1990				0.0392	0.0025	***
	before 1980				reference category		
FDA priority status (priority)		0.0088	0.002	***	0.0141	0.0020	***
Promotional activity (samples)			-			-	
Share of days supplied via mail-order pharmacy (mail)		0.3272	0.003	***	0.3285	0.0034	***
Copayment / day (copay_day)		-0.0347	0.001	***	-0.0341	0.0009	***
Fixed effect patient			✓			✓	
Fixed effect therapeutic class			✓			✓	
Fixed effect year started therapy			✓			✓	

****:p<0.0001; **:p<0.01; *<0.05; FDA: Food and Drug Administration*

b) Regressions by major health conditions

	Hypertension (Model 3)	*Hyper-cholesterolemia (Model 4)*	*Diabetes (Model 5)*	*Hypothyroidism (Model 6)*	*Osteoporosis (Model 7)*
FDA approval year (FDA_year)	0.0034***<br>(0.0003)	0.0037***<br>(0.0003)	0.0023***<br>(0.0004)	0.0038***<br>(0.0005)	0.0037***<br>(0.0006)
FDA priority status (priority%)	0.0360***<br>(0.0058)	0.0146*<br>(0.0062)	0.0157<br>(0.0000)	0.0337**<br>(0.0120)	0.0312*<br>(0.0140)
Promotional activity (samples)	-	-	-	-	-
Share mail-order prescriptions (mail%)	0.3687***<br>(0.0122)	0.3193***<br>(0.0129)	0.3317***<br>(0.0207)	0.3474***<br>(0.0261)	0.3669***<br>(0.0318)
Copayment / day (copay_day)	-0.0250***<br>(0.0038)	-0.0286***<br>(0.0040)	-0.0672***<br>(0.0080)	-0.0089<br>(0.0062)	-0.0192<br>(0.0122)
Person-level Fixed Effects	yes	yes	yes	yes	yes
Therapeutic Class Fixed Effects	yes	yes	yes	yes	yes
Year started therapy	yes	yes	yes	yes	yes
N (patient - drug class combinations)					
N (patients)					

****:p<0.0001; **:p<0.01; *<0.05; FDA: Food and Drug Administration*

SUPPLEMENTARY MATERIAL 4 – ROBUSTNESS

Log-odds of MPR as outcome variable

Since the MPR is bounded between zero and one, we assess the robustness of our results when we transform the level of MPR to the level of log odds (i.e. $\log\left(\frac{\overline{MPR_{ic}}}{1-\overline{MPR_{ic}}}\right)$) as dependent variable in the regression analyses.

a) Pooled regressions

		Model 1 (drug vintage continous)	*Model 2 (drug vintage by decades)*
Intercept			
FDA approval year (FDA_year)		0.0087*** (0.0003)	
FDA approval year (categorical)	*later than 2000*		0.2696*** (0.0158)
	between 1991 and 2000		0.3205*** (0.0117)
	between 1981 and 1990		0.1471*** (0.0114)
	before 1980		
FDA priority status (priority%)		0.0124 (0.0087)	0.0378*** (0.0091)
Promotional activity (samples%)		0.0063*** (0.0010)	0.0071*** (0.0010)
Share of days supplied via mail-order pharmacy (mail)		1.4210*** (0.0157)	1.4245*** (0.0157)
Copayment / day (copay_day)		-0.1513*** (0.0042)	-0.1468*** (0.0042)
Fixed effect patient		yes	yes
Fixed effect therapeutic class		yes	yes
Fixed effect year started therapy		yes	yes

****:p<0.0001; **:p<0.01; *<0.05; FDA: Food and Drug Administration; Standard errors are in parantheses.*

b) Regressions by major health conditions

	Hypertension (Model 3)	*Hyper-cholesterolemia (Model 4)*	*Diabetes (Model 5)*	*Hypothyroidism (Model 6)*	*Osteoporosis (Model 7)*
FDA approval year (FDA_year)	0.0115*** (0.0022)	0.0107*** (0.0024)	0.0058 (0.0041)	0.0375*** (0.0050)	0.0255*** (0.0038)
FDA priority status (priority)	0.0064 (0.0487)	-0.1363** (0.0521)	-0.1309 (0.0897)	0.1939 (0.1255)	0.3860*** (0.0911)
Promotional activity (log of samples)	-0.01576417* (0.00787781)	0.00713703 (0.00876797)	-0.03155182* (0.01483920)	-0.023479574 (0.01899987)	-0.07860113*** (0.01713624)
Share mail-order prescriptions (mail)	1.7076*** (0.1043)	2.1687*** (0.1091)	0.7274** (0.1921)	2.5085*** (0.2589)	1.4017*** (0.1764)
Copayment / day (copay_day)	-0.1661*** (0.0280)	-0.1999*** (0.0313)	-0.3506*** (0.0706)	-0.1725 (0.1002)	-0.1409 (0.0776)
Person-level Fixed Effects	yes	yes	yes	yes	yes
Therapeutic Class Fixed Effects	yes	yes	yes	yes	yes
Year started therapy	yes	yes	yes	yes	yes
N (patient - drug class combinations)	60,525	49,431	23,249	16,778	9,757
N (patients)	10,741	10,061	3,455	3,232	1,981

****:p<0.0001; **:p<0.01; *<0.05; FDA: Food and Drug Administration; Standard errors are in parantheses.*

Subgroup analyses

Patient subgroups with switching behavior or single brand use – pooled regressions

	Model 1 (multiple substances in same class)	*Model 1 (one manufacturer brand only)*
**Product quality**		
FDA approval year (FDA_year)	0.0020*** (0.0002)	0.0033*** (0.0001)
FDA priority status (priority%)	0.0193** (0.0058)	0.0079** (0.0026)
Situational factors		
Promotional activity (samples)	0.0031*** (0.0010)	0.0034*** (0.0000)
Share of days supplied via mail-order pharmacy (mail%)	0.3358*** (0.0113)	0.3177*** (0.0046)
Copayment / day (copay_day)	-0.0232*** (0.0031)	-0.0287*** (0.0011)
Fixed effect patient	yes	
Fixed effect therapeutic class	yes	
Fixed effect year started therapy	yes	
R-Squared	0.887	0.767
Root MSE	0.152	0.210
N	40,752	158,988

****:p<0.0001; **:p<0.01; *<0.05; FDA: Food and Drug Administration; Standard errors are in parantheses.*

SUPPLEMENTARY MATERIAL 5 – PANEL REGRESSION

To assess whether our results hold across different research designs, we estimated models using aggregate longitudinal data at the level of a drug class. These models enable us to answer the question, has adherence increased more in drug classes in which there has been more innovation? Models using aggregate longitudinal data are unlikely to be biased by unobserved patient heterogeneity [25]. We aggregated all data to the level of drug class c in year t and used the full time range available, i.e. 1996 to 2013. We estimated the following linear panel regression model:

$$adherence_{ct} = \beta_1 RX\_vintage_{ct} + \mathbf{\Pi}\boldsymbol{X_{ct}} + \alpha_c + \delta_t + \varepsilon_{ct},$$

weighted by N_{ct}, i.e. the number of patients in drug class c at time t. $RX\_vintage_{ct}$ is the weighted mean FDA approval year of drugs in therapeutic class c dispensed in year t, weighted by the number of days supplied for each molecule, $\mathbf{\Pi}\boldsymbol{X_{ct}}$ is the vector of covariates, α_c a fixed effect for therapeutic class c, δ_t a time fixed effect for the year and, ε_{ct} is the disturbance term.

The results of the research design that uses aggregate longitudinal data confirm the positive relationship between drug vintage and medication adherence (see table below). Pooling across all therapeutic classes, conditions and patients in different stages of their drug therapy, we observed a smaller, but significant effect of FDA approval year (i.e. 0.011 compared to 0.02 in model 1) on the log-odds of MPR.

Results of panel regressions

Variable	*estimate*	*p-value*	*estimate*	*p-value*	*estimate*	*p-value*	*estimate*	*p-value*	*estimate*	*p-value*	*estimate*	*p-value*	*estimate*	*p-value*	*estimate*	*p-value*	*estimate*	*p-value*
Intercept	-23.445	0.009	-1.633	0.002	-1.653	0.002	-1.639	0.001	-23.817	0.010	-19.446	0.054	-24.515	0.010	-23.362	0.007	-19.527	0.050
FDA approval year	0.011	0.013							0.011	0.015	0.009	0.073	0.012	0.016	0.011	0.011	0.009	0.066
FDA approval year post 1980			0.506	0.029														
FDA approval year post 1990					0.408	0.011												
FDA approval year post 2000							0.398	0.039										
FDA priority status			0.210	0.211	0.279	0.080	0.333	0.061			0.218	0.213					0.226	0.175
Metric quantity / day (log-scale)															-0.136	0.134		
Share extended / delayed - disease formulations													0.159	0.342				
Co-pay / day (log scale)									-0.078	0.256			-0.085	0.222	-0.092	0.166		
ln_enrolid			0.013	0.849	0.022	0.737	0.020	0.760									0.019	0.771
year levels	Included		Included		Included		Included		Included		Included		Included		Included		Included	
therapeutic class levels	Included		Included		Included		Included		Included		Included		Included		Included		Included	
